# 5-O-Methylvisammioside inhibits HMGB1-induced Angiogenesis of hepatocellular carcinoma through RAGE/MEK/ERK signaling pathway

**DOI:** 10.1371/journal.pone.0322056

**Published:** 2025-05-05

**Authors:** Wenyue Hou, Ting Zou, Yichao Yan, Yaolong Zhuang, Shaomei Gao, Huijun Ju, Fei Yao, Qin Yuan, Liang Zhou, Guoqiang Liang, Xiudao Song, Lurong Zhang

**Affiliations:** 1 Suzhou TCM Hospital Affiliated to Nanjing University of Chinese Medicine, Suzhou, China; 2 College of Life Sciences, Xuzhou Medical University, Xuzhou, China; 3 Suzhou Academy of Wu Men Chinese Medicine, Suzhou, China; Rutgers: Rutgers The State University of New Jersey, UNITED STATES OF AMERICA

## Abstract

5-O-Methylvisammioside (5OMV), a flavonol compound derived from the traditional Chinese medicine plant *Saposhnikovia divaricat*, has been shown to inhibit vasospasm induced by High Mobility Group Box 1 (HMGB1) protein. However, its therapeutic potential and molecular mechanisms in HMGB1-induced tumor angiogenesis remain unexplored. Through comprehensive *in vitro* assays, we demonstrated that 5OMV significantly attenuates HMGB1-induced proliferation, migration, tube formation, and angiogenic activity in human umbilical vein endothelial cells (HUVECs). Parallel *in vivo* studies using an orthotopic hepatocellular carcinoma model in C57BL/6 mice revealed that 5OMV treatment markedly reduced tumor progression and microvascular density. Mechanistic studies identified that 5OMV downregulates both total and phosphorylated forms of RAGE, MEK, and ERK in HUVECs and tumor tissues. These findings collectively establish that 5OMV exerts anti-tumor effects in hepatocellular carcinoma through targeted modulation of the HMGB1/RAGE/MEK/ERK signaling axis.

## Introduction

Hepatocellular carcinoma (HCC), the predominant form of primary liver malignancy, accounting for over 90% of cases, ranks as the fourth most prevalent malignant tumor and the second leading cause of cancer-related mortality in China [1]. The clinical challenge stems from the typically asymptomatic nature of early-stage HCC, which leads to frequent diagnosis at advanced stages. Current therapeutic interventions for advanced HCC demonstrate limited efficacy, with a five-year survival rate of approximately 18% [2]. This prognostic constraint underscores the imperative for developing novel and effective treatment modalities as a paramount objective in contemporary HCC research.

Sustained angiogenesis, recognized as one of the 14 hallmarks of cancer, is typically characterized by excessive vascular proliferation and significant vascular abnormalities. Tumor neovessels represent a type of malformed vasculature that interconnects with adjacent normal blood vessels, exhibiting distinct pathophysiological characteristics such as increased luminal permeability and compromised oxygen transport capacity compared to normal vasculature. HCC, a prototypical hypervascular tumor, demonstrates aggressive biological behaviors closely associated with its abundant tumor vascular network [3]. Clinically approved anti-angiogenic therapeutic agents, including sorafenib, regorafenib, and lenvatinib, have been demonstrated to significantly improve survival rates in patients with advanced HCC [4–6]. Therefore, targeting angiogenesis in HCC remains a crucial therapeutic strategy to delay disease progression in intermediate and advanced-stage HCC.

The process of angiogenesis in HCC is intricate. Previous research has highlighted that HCC cells secrete angiogenic factors, such as vascular endothelial growth factor (VEGF), which increases tumor capillary permeability and promotes the proliferation of endothelial cells within the tumor. Recent studies have further illuminated the close interplay between tumor angiogenesis and the immune microenvironment [7–9]. In HCC, angiogenesis is modulated by inflammatory factors present in the tumor microenvironment, such as high-mobility group box 1 protein (HMGB1).

HMGB1, a non-histone chromatin-binding protein, originates from extracellular sources: (1) active secretion by monocytes, macrophages, and dendritic cells; (2) passive release from the nucleus during pathological processes such as tissue necrosis; and (3) macrophage-mediated secretion HMGB1 after phagocytosis of necrotic cells. Clinical evidence indicates that elevated HMGB1 expression in HCC tissues strongly correlates with tumor angiogenesis [10]. Mechanistically, HMGB1 directly stimulates endothelial cells to promote vascular tube formation. Antibody-mediated inhibition of HMGB1 significantly suppresses angiogenesis, thereby attenuating HCC progression [11]. These findings demonstrate that HMGB1 overexpression in HCC drives vascular proliferation and accelerates disease progression. Therefore, therapeutic targeting of HMGB1-mediated angiogenic signaling may constitute a novel strategy for liver cancer treatment.

5-O-Methylvisammioside (5OMV), a flavonol glycoside compound, is a primary bioactive constituent of *Saposhnikovia divaricata (Turcz.) Schischk*., a dicotyledonous medicinal plant widely used in traditional Chinese medicine [12]. Our prior experimental data have revealed the anti-HCC properties of Radix Saposhnikoviae (Fangfeng), with the classical formulation Yupingfeng San(YPFS)—containing Saposhnikoviae Radix as a principal component—demonstrating potent anti-angiogenic effects against HCC vasculature [13]. Importantly, 5OMV has been established as a phytochemical marker for YPFS quality control [14]. Mechanistically, preclinical studies have documented that 5OMV administration significantly suppresses subarachnoid hemorrhage in rat models by inhibiting the HMGB1 signaling axis, concomitant with improvement in vascular homeostasis [15]. Nevertheless, the therapeutic potential and molecular mechanisms underlying 5OMV-mediated inhibition of HMGB1-induced hepatic neovascularization in HCC remain to be elucidated.[16].

As a multi-receptor ligand, HMGB1 binds to various receptors, including Toll-like receptors and the receptor for advanced glycation end-products (RAGE). RAGE is recognized as the most critical and earliest identified receptor for HMGB1, exhibiting exceptionally high affinity. Although RAGE is typically expressed at low levels in normal tissues, HMGB1 binding to RAGE can induce an upregulation of RAGE expression [17]. Recent research has elucidated the roles of both HMGB1 and RAGE in the progression, invasion, and metastasis of various tumors [15]. Upon binding to RAGE on endothelial cells, HMGB1 initiates a critical signaling cascade through sequential activation of mitogen-activated protein kinase kinase (MEK) and its downstream effector extracellular signal-regulated kinase (ERK). This phosphorylation-driven signaling axis stimulates endothelial cell proliferation and promotes pathological neovascularization. The use of a neutralizing antibody to block RAGE has been shown to inhibit HMGB1-induced angiogenesis *in vivo* and endothelial cell proliferation *in vitro* [18]. This underscores the regulatory mechanism by which HMGB1 promotes angiogenesis in hepatocellular carcinoma, closely tied to the RAGE/MEK/ERK signaling pathway.

In summary, our study demonstrates that 5OMV exerts anti-hepatocellular carcinoma effects by inhibiting HMGB1-induced angiogenesis. This conclusion is supported by findings from both *in vitro* models involving HMGB1-induced endothelial cell and vascular tissue proliferation and a C57BL/6J in situ transplantation mouse model. Furthermore, we elucidated that the inhibitory effect of 5OMV on HMGB1-induced angiogenesis is mediated through the RAGE/MEK/ERK signaling pathway. These findings provide a strong rationale for considering 5OMV as a promising natural compound for the development of anti-hepatocellular carcinoma agents and offer compelling evidence supporting HMGB1 as a potential target for anti-angiogenesis therapy.

## Materials and methods

### Chemical

5-O-Methylvisammioside (5OMV) was obtained from PureOne (Cat# 22082404, Shanghai, China). High Mobility Group Box1 (HMGB1) protein was sourced from Solarbio (Cat# P00077, Beijing, China).

### Moral statement

The research protocol and animal utilization in this study were approved by the Ethics Committee of Suzhou Hospital of Traditional Chinese Medicine (Ethics Research Lot No. 047, 2022). All animal care and experiments were conducted in strict accordance with institutional guidelines for the care and use of experimental animals. The researchers were trained in animal care or handling.

All laboratory animals were kept in ventilated cages under standard laboratory conditions, with free access to food and water. Room temperatures were maintained between 18–22°C to ensure their comfort and minimize stress-related effects.

### Cell lines and animals

Human umbilical vein endothelial cells (HUVECs; ATCC CRL-1730) and mouse hepatoma Hepa1–6 cells (Soochow University Cell Bank) were cultured in complete medium (Dulbecco’s Modified Eagle Medium [DMEM] [Cat# 2463414, Gibco, USA] supplemented with 10% fetal bovine serum [FBS] [Cat# 11011-8611, TIANHANG, China]) under standard conditions (37°C, 5% CO_2_, humidified atmosphere).

Twenty-five male C57BL/6 mice (8 weeks, 20 ± 2 g; Hangzhou Ziyuan Laboratory Animal Technology Co., Ltd., production license number: SCXK [Zhe] 2019–0004; Quality Certificate number: 20230220Abbz0105000927) and ten male SD rats (6–8 weeks, 200 ± 20 g; Zhaoyan New Drug Research Center; License number: SCXK [Su] 2018–0006; Quality Certificate number: 202268776) were included. All animals were maintained under specific pathogen-free conditions.

The 25-day experimental period involved daily monitoring of body weight, clinical signs (fur condition, activity levels), and welfare indicators (body temperature), with predefined humane endpoints requiring euthanasia if animals exhibited >15% body weight loss, behavioral abnormalities (social withdrawal, stereotypy), hypothermia (<34°C), or hyperthermia (>39°C). No animals met these criteria, and thus no premature euthanasia was necessary during the study.

### Toxicity and CELL proliferation assay

HUVECs were seeded in 96-well plates (4 × 10^3^ cells/well) and treated with indicated compounds for 24 h. After medium aspiration, MTT solution (0.5 mg/mL) (Cat# 70080125, biosharp, China) was added and incubated for 4 h. Formazan crystals were dissolved with DMSO (Cat# 20150906, DAMAO, China), and absorbance at 490 nm was measured using a microplate reader. Cell proliferation was normalized to untreated control. The data are shown as a percentage of the control. All experimental assays were independently repeated in triplicate.

### Wound healing assay

HUVECs were seeded in 6-well plates 2 × 10^5^ cells/well and cultured to confluence. Uniform linear wounds were created using a sterile pipette tip, followed by PBS washes to remove cellular debris. Phase-contrast images of wounds were captured at 0 h and 24 h. Cell migration was quantified in ImageJ by calculating: Percentage of Wound closure was calculated as (*A*_*0*_ - *A*_*t*_)/ *A*_*0* _× 100%, where *A*_*0*_ and *A*_*t*_ represent the wound area at 0 h and 24 h, respectively [16]. All experimental assays were independently repeated in triplicate.

### Transwell migration assay

A Transwell migration assay was performed using 6-well plates with 8 μm pore chambers. Endothelial cells (1 × 10⁴ cells/well in serum-free DMEM) were seeded into the upper chambers, while the lower chambers contained 600 μL DMEM supplemented with 20% FBS as a chemoattractant. Experimental groups received test compounds in the upper chambers (final volume 100 μL), whereas blank controls received a complete medium. After 24 h incubation, non-migrated cells were removed by gentle cotton swab abrasion. Migrated cells were fixed with 4% paraformaldehyde (15 min), stained with 0.1% crystal violet (15 min) (Cat# 112520210514, Beyotime, China), and imaged under phase-contrast microscopy (3 random fields/membrane). For quantitative analysis, stained cells were solubilized with 10% acetic acid (200 μL/well), and absorbance at 570 nm was measured using a microplate reader. The data are shown as a percentage of the control. All experimental assays were independently repeated in triplicate.

### Tube formation assay

A 96-well tube formation assay was performed by coating each well with 50 μL Matrigel (Cat# 354234, Corning, USA) (diluted 1:1 in serum-free medium) and incubating at 37 °C for 30 min. HUVECs (1 × 10⁵ cells/well) were seeded onto the polymerized matrix and treated with designated compounds for 16 h. Three random fields per well were selected and imaged using phase-contrast microscopy. The number of intact tubular structures with visible lumens was manually counted. All experimental assays were independently repeated in triplicate.

### Immunofluorescence

For cellular immunofluorescence, cells (1–2 × 10⁴) were cultured on coverslips in 6-well plates (37°C, 5% CO₂), fixed with 4% paraformaldehyde (15 min), treated with 3% H₂O₂ (10 min), and blocked with 5% species-matched serum. Tissues were processed as formalin-fixed paraffin-embedded sections with antigen retrieval (citrate buffer, 95°C, 20 min). Both cell and tissue samples were incubated with RAGE primary antibody (1:1000, 4°C overnight) followed by species-specific secondary antibody (1:500, 2 h), counterstained with DAPI, and imaged by confocal microscopy. All experimental assays were independently repeated in triplicate.

### Aortic ring assay

Following one week of acclimatization, rats were euthanized via CO₂ inhalation (AVMA guidelines), with death confirmed by cardiopulmonary arrest. Abdominal aortic segments (1 cm) were excised and rinsed in DMEM.

For the aortic ring assay, 96-well plates were coated with 50 μL Matrigel (diluted 1:1 in PBS) and polymerized (37°C, 30 min). Aortic rings (1 mm) were dissected under sterile conditions, embedded in Matrigel, and cultured in an endothelial growth medium (37°C, 5% CO₂). Test compounds were administered at indicated concentrations from day 3 onward, with the medium refreshed every 72 h. Microvascular sprouting was quantified daily (days 3–14) by counting branch points in three random fields/wells using phase-contrast microscopy [16]. All experimental assays were independently repeated in triplicate.

### Animal model

Before orthotopic tumor implantation, mice were anesthetized via intraperitoneal injection of 0.2 mL anesthetic (0.8 g tribromoethanol (Cat# E2210145, Aladdin, China), 0.5 mL tert-amyl alcohol (Cat# C2223052, Aladdin, China), 39.5 mL saline). A midline laparotomy was performed to inject Hepa1–6 cells into the left hepatic lobe. The abdominal wall was sutured and treated with penicillin (8 × 10⁵ IU). Twenty-five male C57BL/6 mice with confirmed tumors were randomized into: Model group (vehicle control), 5OMV groups (12.5, 25, 50 mg/kg), and Sorafenib group (1 mg/kg, positive control). After 14 days of treatment, euthanasia was performed via CO₂ inhalation, with death confirmed by cardiopulmonary arrest. Liver and tumor tissues were collected. All procedures adhered to AVMA guidelines.

### IHC evaluation of tumor microvascular density

Tissue sections were deparaffinized, subjected to antigen retrieval (citrate buffer, pH 6.0, 95°C, 20 min), and treated with 3% H₂O₂ to block endogenous peroxidases. After serum blocking (5% normal serum, 1 h), sections were incubated overnight at 4°C with CD31 primary antibody (1:500), followed by species-matched HRP-conjugated secondary antibody (1:1000, 1 h RT). Staining was developed with DAB, counterstained with hematoxylin, dehydrated, and mounted. Three random fields per section were analyzed at 400 × magnification. The H-score was calculated to quantify staining intensity. All experimental assays were independently repeated in triplicate.

The H-scores were calculated as follows: H-score=∑ (pi × i) = (percentage of weak intensity) × 1 + (percentage of moderate intensity) × 2 + (percentage of strong intensity) × 3.

(where pi represents the percentage of cells exhibiting a particular intensity (weak, moderate, or strong), and i represents the intensity level (1 for weak, 2 for moderate, and 3 for strong)

### ELISA assay

HMGB1 (Cat# 102722221123, Beyotime, China) and VEGF (Cat# A28320323, MULTISCIENCES, China) concentrations in tissue homogenates were quantified using commercial ELISA kits according to the manufacturer’s instructions. Tissue lysates were centrifuged (12,000 × g, 10 min, 4°C) and supernatants analyzed in triplicate. All experimental assays were independently repeated in triplicate.

### Western blotting

Proteins were extracted using RIPA buffer, separated by 10% SDS-PAGE (80 V, 120 min), and transferred to PVDF membranes (100 V, 90 min). Membranes were blocked with 5% non-fat milk/TBST (2 h, RT) and incubated with primary antibodies (4°C, 16 h): RAGE (1:2000, Cat# ab37647, Abcam, UK); MEK1/2 (1:2000, Cat# CST #4694, Cell Signaling Technology, USA); p-MEK1/2 (Ser221) (1:2000, Cat# CST #2338, Cell Signaling Technology, USA); p-ERK1/2 (Thr202/Tyr204) (1:2000, Cat# CST #4370, Cell Signaling Technology, USA); ERK1/2 (1:2000, Cat# CST #9102, Cell Signaling Technology, USA); HMGB1 (1:2000, Cat# CST #3935, Cell Signaling Technology, USA). After TBST washes, HRP-conjugated secondary antibodies (1:5000, RT, 2 h) were applied. Chemiluminescent signals were quantified using Image J with β-actin (1:2000, Cat# 441430NJ-I4222006, Engibody, USA) normalization. All experimental assays were independently repeated in triplicate.

### Molecular docking

Molecular docking was performed using the molecular simulation software CB Dock 2 (https://cadd.labshare.cn/cb-dock2/php/index.php). The protein crystal structures were obtained from the Protein Data Bank (http://www.rcsb.org/pdb/home/home.do) database. Appropriate parameters were set to obtain the protein-ligand complexes [19].

### Statistical analysis

Data analysis was performed using GraphPad Prism 8.0. Quantitative results are presented as mean ± standard deviation (SD). Between-group comparisons were analyzed using two-tailed Student’s t-tests. Statistical significance was defined as *P* < 0.05.

## Results

### 5OMV inhibits HMGB1-induced cell proliferation in HUVECs

**Fig 1:**
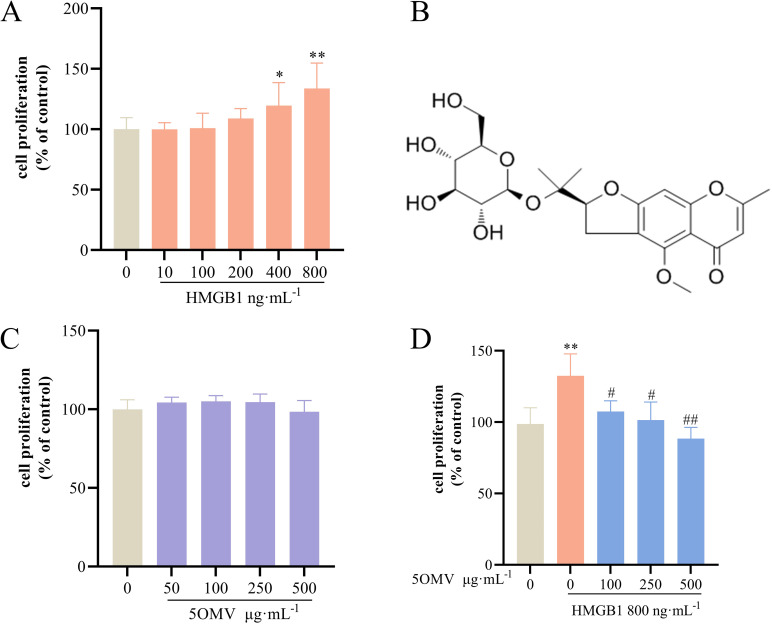
5OMV Inhibits HMGB1-Induced Proliferation of HUVECs. MTT assay—a validated method measuring cellular metabolic activity through the reduction of tetrazolium salts (MTT) to water-soluble formazan crystals, where absorbance at 490 nm correlates with viable cell numbers. **(A)** Cell proliferation assays showed that treatment with HMGB1 at concentrations ranging from 10 to 800 ng·ml^−1^ for 24 h resulted in a dose-dependent increase in the proliferation of HUVECs (Vehicle control: complete medium). **(B)** The chemical structure of 5OMV was illustrated. **(C)** Cell proliferation assays indicated that treatment with 5OMV at concentrations ranging from 50 to 500 μg·mL^−1^ for 24 h had no significant effect on the proliferation of HUVECs (Vehicle control: 0.1% DMSO). **(D)** Cell proliferation assays demonstrated that in the model group treated with 800 ng·ml^-1^ HMGB1 for 24 h, and other groups co-treated with 100–500 μg mL^−1^ of 5OMV under the same conditions for 24 h, 5OMV was able to inhibit HMGB1-induced proliferation of HUVECs (Vehicle control: 0.1% DMSO). The data are shown as a percentage of the control. Data are expressed as the mean ± SD (n = 6). Error bars represented the standard deviation of three technical replicates. Mean values showing significant differences between the control group and the HMGB1-treated group are indicated by *(*P* < 0.05) or ** (*P* < 0.01). Mean values showing significant difference between the HMGB1-treated group and the 5OMV-treated group are denoted by ^#^ (*P* < 0.05) or ^##^ (*P* < 0.01). Abbreviation: 5OMV 5-O-Methylvisammioside; HMGB1 High Mobility Group Box 1; HUVECs human umbilical vein endothelial cells; MTT Methylthiazolyldiphenyl-tetrazolium bromide; DMSO Dimethyl sulfoxide.

To evaluate HMGB1’s role in angiogenesis, we hypothesized that HMGB1 stimulates endothelial cell proliferation. In this experiment, we employed the MTT assay, a widely used method for assessing cell proliferation by measuring cellular metabolic activity to determine viable cell numbers. The principle involves the reduction of MTT (3-(4,5-dimethylthiazol-2-yl)-2,5-diphenyltetrazolium bromide) by mitochondrial dehydrogenases in viable cells to water-soluble formazan crystals. The amount of formazan produced is directly proportional to the number of metabolically active cells. Cell proliferation was quantified by measuring formazan absorbance at 490 nm using a microplate reader. we demonstrated dose-dependent proliferation of HUVECs exposed to HMGB1 (0–800 ng/mL, 24 h) [20]. Maximal proliferation (33.69% ± 21.15%, *P* < 0.01 vs. untreated control) was observed at 800 ng/mL (Fig 1A), establishing this concentration as the model group for subsequent studies. The chemical structure of 5OMV is shown in Fig 1B. To assess 5OMV’s cytotoxicity, HUVECs were treated with 5OMV (0–500 μg/mL, 24 h). No significant reduction in cell number was observed (*P* > 0.05 vs. control; Fig 1C), confirming its safety within this therapeutic range. To examine the proliferative and functional effects of 5OMV on HUVECs induced by HMGB1, HMGB1-stimulated HUVECs (800 ng/mL, 24 h) were co-treated with 5OMV (0–500 μg/mL). Dose-dependent suppression of proliferation was observed (Fig 1D). The results demonstrate that 5OMV inhibits HMGB1-induced proliferation of HUVECs.

### 5OMV inhibits HMGB1-induced cell migration and tube formation in HUVECs

**Fig 2:**
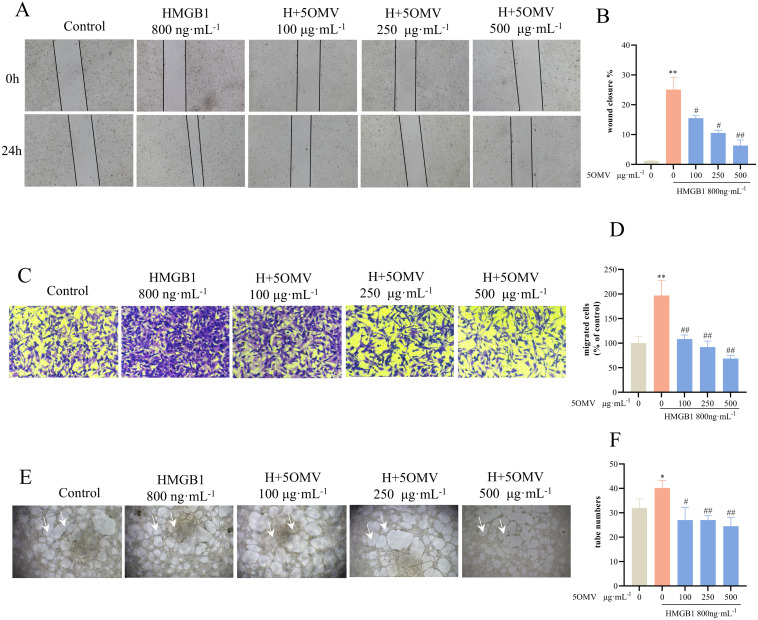
5OMV Inhibits HMGB1-Induced Tube Formation in HUVECs. Representative images **(A)** of the wound healing assay (captured at 0 h and 24 h post-scratching) demonstrated that co-treatment with 5OMV (100, 250, and 500 μg mL^−1^) and HMGB1 (800 ng mL^−1^) significantly attenuated HUVECs migration. Quantitative analysis **(B)** revealed dose-dependent inhibition of wound closure (Vehicle control: 0.1% DMSO). **(C)** Representative images of the Transwell migration assay (24 h) demonstrated that co-treatment with 5OMV (100, 250, and 500 μg mL^−1^) and HMGB1 (800 ng mL^−1^) significantly inhibited HUVECs migration. Quantitative analysis **(D)** revealed a reduction in migrated cells compared to the HMGB1-only group, confirming 5OMV’s dose-dependent anti-migratory effect (Vehicle control: 0.1% DMSO). The data are shown as a percentage of the control. Representative images **(E)** of the tube formation assay (24 h post-treatment) showed that 5OMV (100, 250, and 500 μg mL^−1^) combined with HMGB1 (800 ng mL^−1^) disrupted capillary-like network formation in HUVECs (white arrows indicate intact tubular structures). Quantitative analysis **(F)** demonstrated a decrease in total tube numbers compared to HMGB1 stimulation alone, highlighting 5OMV’s potent anti-angiogenic activity (Vehicle control: 0.1% DMSO). The number of cells in each well/membrane was randomly counted in three microscopic fields (magnification 100×). The data were presented as mean ± SD (n = 3). Error bars represented the standard deviation of three technical replicates. Mean values showing significant differences between the control group and the HMGB1-treated group are indicated by *(*P* < 0.05) or ** (*P* < 0.01). Mean values showing significant difference between the HMGB1-treated group and the 5OMV-treated group are denoted by ^#^ (*P* < 0.05) or ^##^ (*P* < 0.01). Abbreviation: 5OMV 5-O-Methylvisammioside; HMGB1 High Mobility Group Box 1; HUVECs human umbilical vein endothelial cells.

Angiogenesis encompasses proliferation, migration, and tubulogenesis. To assess 5OMV’s effects on HMGB1-induced endothelial migration, wound healing assays demonstrated that HMGB1 (800 ng/mL) significantly reduced wound width compared to controls (*P* < 0.01; Fig 2A-B), indicating enhanced migration. 5OMV treatment attenuated this effect, with reduced wound closure (*P* < 0.01 vs. HMGB1-only). To exclude proliferation interference, Transwell assays confirmed HMGB1 increased migrated cell counts (*P* < 0.01 vs. control; Fig 2C–D), while 5OMV suppressed this enhancement (*P* < 0.01). In tube formation assays, HMGB1 promoted robust capillary networks (*P* < 0.01 vs. control; Fig 2E–F), whereas 5OMV disrupted tube integrity and reduced branch points (*P* < 0.05, *P* < 0.01). Collectively, 5OMV inhibits HMGB1-induced angiogenesis by targeting migration and tubulogenesis.

### 5OMV inhibits HMGB1-induced microvascular branch formation in rat abdominal aorta

**Fig 3:**
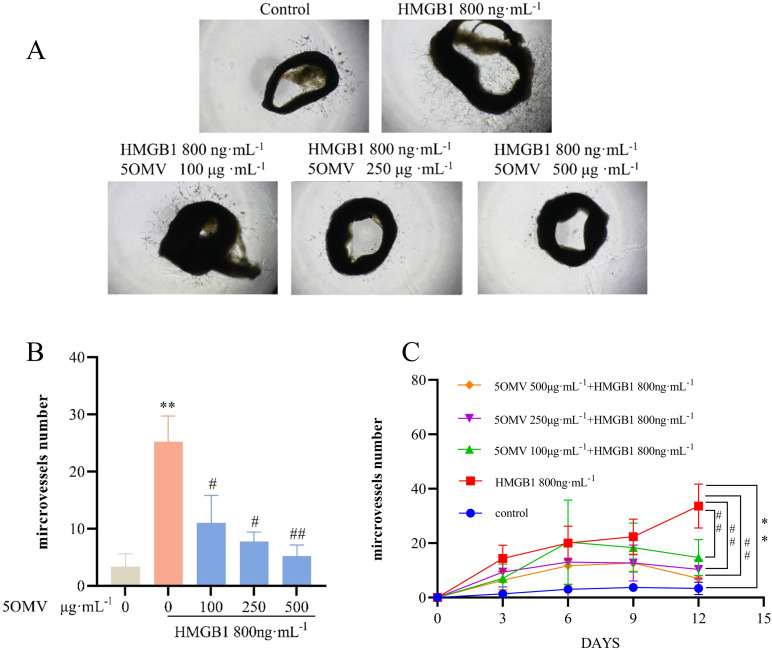
5OMV Inhibits HMGB1-Induced Microvascular Branch Formation in Rat Abdominal Aortic Rings. Representative images **(A)** and the quantitative analysis of microvascular branching in the aortic ring in **(B)** indicated that treatment with 5OMV at concentrations of 100, 250, and 500 μg·mL^−1^, in conjunction with 800 ng·ml^−1^ of HMGB1 for 24 h, significantly reduced the number of microvessels in HUVECs (Vehicle control: 0.1% DMSO). **(C)** After 12 days of drug treatment with the rat aortic ring culture, the number of microvessel sprouts gradually decreased with increasing concentrations of 5OMV. The number of microvascular branches in each well was observed under the microscope (magnification 100×). The data were presented as mean ± SD (n = 3). Error bars represented the standard deviation of three technical replicates. Mean values showing significant differences between the control group and the HMGB1-treated group are indicated by *(*P* < 0.05) or ** (*P* < 0.01). Mean values showing significant difference between the HMGB1-treated group and the 5OMV-treated group are denoted by ^#^ (*P* < 0.05) or ^##^ (*P* < 0.01). Abbreviation: 5OMV 5-O-Methylvisammioside; HMGB1 High Mobility Group Box 1; HUVECs human umbilical vein endothelial cells.

To further validate 5OMV’s inhibitory effects on HMGB1-induced angiogenesis, ex vivo rat aortic ring assays were performed. Aortic rings embedded in matrix gel demonstrated increased microvascular branch formation around HMGB1-treated rings compared to controls (*P* < 0.01; Fig 3A–B). Co-treatment with 5OMV significantly reduced microvascular sprouting in a dose-dependent manner (*P* < 0.05, *P* < 0.01 vs. HMGB1-only). Longitudinal observation revealed sustained sprouting in HMGB1-treated rings over time, whereas 5OMV treatment induced progressive atrophy of vascular buds, with reduced sprout counts evident after one week (Fig 3C). These results confirm that HMGB1 promotes microvascular formation in aortic rings, while 5OMV suppresses this process by reducing microvascular density and sprouting.

### 5OMV anti-tumor effects by inhibiting tumor angiogenesis

**Fig 4:**
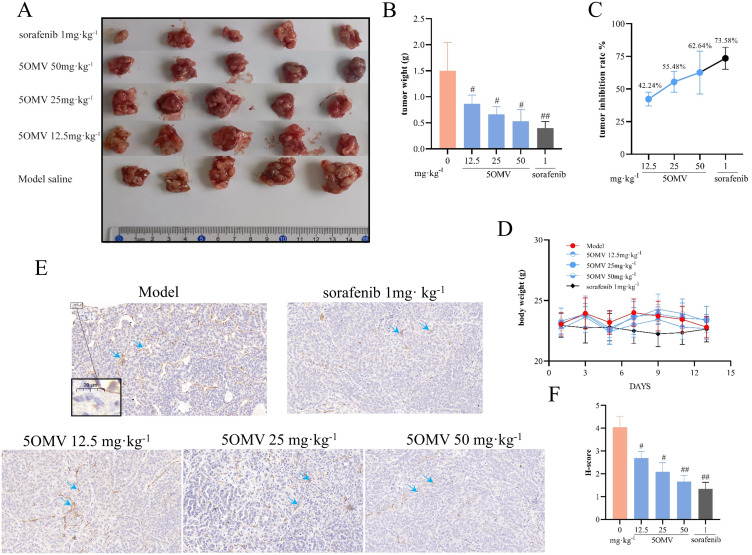
5OMV Inhibits Tumor Growth and Tumor Angiogenesis in an In Situ Hepatocellular Carcinoma Mouse Model. **(A)** On day 14 after treatment, liver tumor tissues from each group of mice were collected. **(B)** The weights of liver tumor tissues from different groups of mice were measured. **(C)** The tumor suppression rates for different groups compared to the model group. **(D)** The body weight changes of liver cancer mice were monitored over the 14-day treatment period. Representative images **(E)** and **(F)** showed the CD31 immunohistochemical analysis of mouse liver tumor tissues, with the brown-stained areas indicating microvessels, as indicated by the blue arrows (scale bar = 20 μm). The data were presented as mean ± SD (n = 3), with error bars representing the standard deviation of three technical replicates. Mean values showing significant differences between the model group and the other groups are denoted by ^#^ (*P* < 0.05) or ^##^ (*P* < 0.01). Abbreviation: 5OMV 5-O-Methylvisammioside; CD31 platelet endothelial cell adhesion molecule-1.

To evaluate whether 5OMV exerts anti-HCC effects through angiogenesis inhibition, in vivo studies were conducted. Tumor-bearing mice exhibited no significant changes in body weight across groups during the experimental period (Fig 4D), suggesting that 5OMV may have low toxicity in tumor-bearing mice based on body weight monitoring. Tumor weight analysis revealed that 5OMV significantly reduced tumor mass compared to the model group (*P* < 0.05; Fig 4A, B), with tumor inhibition rates of 42.24% ± 7.71%, 55.48% ± 9.13%, and 62.64% ± 14.72%, in respective treatment groups (*P* < 0.05; Fig 4C). Immunohistochemical staining of CD31-labeled microvessel density (MVD) showed that 5OMV treatment significantly decreased H-scores in tumor tissues (*P* < 0.05, *P* < 0.01 vs. model group; Fig 4E, F). These results indicate that 5OMV likely inhibits HCC progression by suppressing tumor angiogenesis.

### 5OMV inhibits HMGB1-induced tumor angiogenesis through the RAGE/MEK/ERK signaling pathway

**Fig 5:**
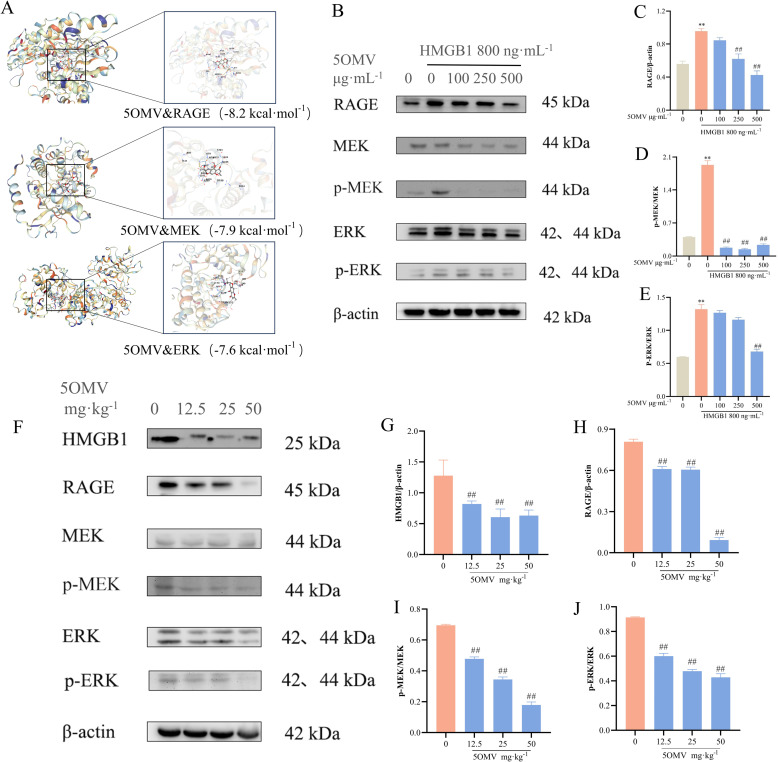
5OMV Inhibits RAGE/MEK/ERK Protein Expression. **(A)** Computational docking simulations revealed the interaction between 5OMV and RAGE/MEK/ERK. **(B)** Representative Western blots and quantitative analyses of **(C)** RAGE/β-actin ratio, **(D)** p-MEK/MEK ratio, and **(E)** p-ERK/ERK ratio demonstrated that 5OMV (100-500 μg·mL^−1^) significantly inhibited HMGB1-induced upregulation of RAGE, p-MEK, and p-ERK in HUVECs. **(F)** Representative blots and quantitative analyses of **(G)** HMGB1/β-actin ratio, **(H)** RAGE/β-actin ratio, **(I)** p-MEK/MEK ratio, and (J) p-ERK/ERK ratio expression in tumor tissues from HCC mice showed that 5OMV (12.5–50 mg kg^−1^) dose-dependently suppressed the elevated levels of HMGB1, RAGE, and ERK phosphorylation observed in the model group. Data were presented as mean ± SD (n = 3). Error bars represented the standard deviation of three technical replicates. Mean values showing significant differences between the control group and the HMGB1-treated group are indicated by *(*P* < 0.05) or ** (*P* < 0.01). Mean values showing significant difference between the model group and the 5OMV-treated group are denoted by ^#^ (*P* < 0.05) or ^##^ (*P* < 0.01). Abbreviation: 5OMV 5-O-Methylvisammioside; HMGB1 High Mobility Group Box 1; HUVECs human umbilical vein endothelial cells; RAGE The Receptor of Advanced Glycation Endproducts; MEK Mitogen-activated extracellular signal-regulated kinase; p-MEK Phospho-Mitogen-activated extracellular signal-regulated kinase; ERK Extracellular regulated protein kinases.

To elucidate the mechanism underlying 5OMV’s anti-angiogenic effects, molecular docking simulations predicted strong interactions between 5OMV and the RAGE/MEK/ERK signaling axis, with binding energies below -5 kcal/mol across three independent docking models (Fig 5A). Structural analysis revealed that 5OMV occupies the ligand-binding cavity of RAGE through hydrogen bonds and hydrophobic interactions. Given the critical role of the RAGE/MEK/ERK pathway in angiogenesis, we assessed its modulation in HMGB1-stimulated HUVECs. HMGB1 significantly upregulated RAGE, MEK, ERK, and their phosphorylated forms (p-MEK, p-ERK) compared to controls (*P* < 0.01; Fig 5B–E). 5OMV treatment reversed these effects, suppressing RAGE, MEK, ERK, p-MEK, and p-ERK expression (*P* < 0.01). Consistent with in vitro findings, tumor tissues from 5OMV-treated mice exhibited significant reductions in RAGE, ERK, p-MEK, and p-ERK levels compared to the model group (*P* < 0.01; Fig 5F–J). These results demonstrate that 5OMV inhibits HMGB1-induced angiogenesis by targeting the RAGE/MEK/ERK signaling pathway.

**Fig 6:**
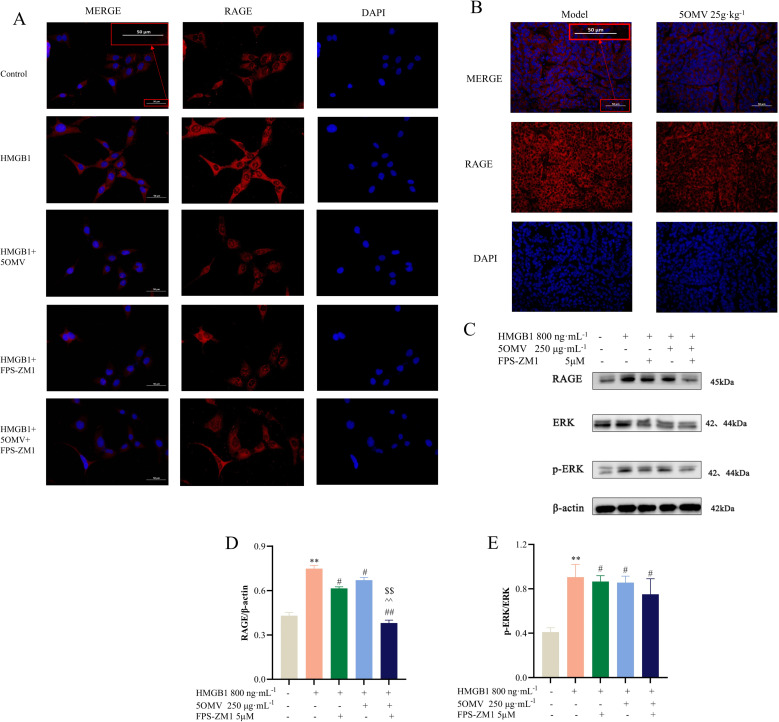
RAGE is a Key Target in HMGB1-Induced Tumor Angiogenesis. **(A)** Representative images of RAGE immunofluorescence staining in HMGB1-induced HUVECs treated with the RAGE inhibitor FPS-ZM1 were shown (scale bar = 50 μm). **(B)** Representative images of RAGE immunofluorescence staining in tumor tissues were shown (scale bar = 50 μm). **(C)** Representative Western blots and quantitative analyses of **(D)** RAGE/β-actin ratio and **(E)** p-ERK/ERK ratio demonstrated that HMGB1 regulates downstream angiogenic signaling proteins via RAGE-dependent activation of the ERK pathway. Data were presented as mean ± SD (n = 3). Error bars represented the standard deviation of three technical replicates. Mean values showing significant differences between the control group and the HMGB1-treated group are indicated by *(*P* < 0.05) or ** (*P* < 0.01). Mean values showing significant differences between the HMGB1-treated group and the other groups are denoted by ^#^ (*P* < 0.05) or ^##^ (*P* < 0.01). Abbreviation: HMGB1 High Mobility Group Box 1; HUVECs human umbilical vein endothelial cells; RAGE The Receptor of Advanced Glycation Endproducts.

To validate the central role of RAGE in 5OMV-mediated suppression of angiogenesis, we examined HMGB1-induced signaling alterations in HUVECs using the RAGE inhibitor FPS-ZM1 [21,22]. Immunofluorescence analysis revealed that HMGB1 markedly increased RAGE expression (red fluorescence) compared to controls, while 5OMV, FPS-ZM1, and their combination significantly reduced RAGE levels in HUVECs (Fig 6A). Consistent with these findings, the inhibitory effect of 5OMV on RAGE expression was also observed in tumor tissues (Fig 6B). Western blotting further demonstrated that HMGB1 upregulated RAGE, ERK, and p-ERK levels (*P* < 0.05, *P* < 0.01 vs. control; Fig 6C–E). In contrast, treatment with FPS-ZM1, 5OMV, or their combination reversed these HMGB1-induced increases (*P* < 0.01 vs. HMGB1 group; Fig 6C–E). Notably, the 5OMV + FPS-ZM1 combination exhibited synergistic suppression of RAGE, ERK, and p-ERK expression compared to either treatment alone (*P* < 0.05, *P* < 0.01; Fig 6C–E). Collectively, these results confirm that HMGB1 promotes angiogenesis via RAGE-dependent activation of the ERK pathway and that pharmacological inhibition of RAGE attenuates 5OMV’s anti-angiogenic effects, establishing RAGE as the critical molecular target through which 5OMV inhibits HMGB1-induced vascularization in HCC.

## Discussion

5-O-Methylvisammioside (5OMV), a flavonol glycoside compound has been recognized for its anti-tumor properties [23]. Prior studies demonstrated that 5OMV attenuates subarachnoid hemorrhage in rats by improving vascular homeostasis through repair mechanisms. To further investigate its antitumor efficacy, we established an orthotopic Hepa1–6 hepatocellular carcinoma model in mice. Tumor microvessel density (MVD) and tumor inhibition rates were quantified, revealing that 5OMV significantly suppressed tumor progression (*P* < 0.01), likely mediated through reduced MVD.Recent studies have identified angiogenesis as one of the 14 hallmarks of tumor progression [24]. This complex physiological process involves degradation of the vascular basement membrane during the activation phase, followed by endothelial cell activation, proliferation, migration, and subsequent reconstruction to form new vascular networks. As a critical focus in anti-tumor research, anti-angiogenesis strategies have garnered significant attention. HMGB1, the most abundant member of the HMGB family, plays multifaceted roles in both physiological and pathological processes. While previous investigations predominantly emphasized its involvement in inflammatory responses, accumulating clinical evidence now highlights its pivotal contributions to tumorigenesis. Experimental studies demonstrate that HMGB1 stimulates endothelial cells to enhance tube formation and induces endothelial cell sprouting from MAE cell aggregates embedded in three-dimensional fibrin gels [17]. Notably, HMGB1 silencing has been shown to suppress tumor growth in murine models [7]. In our study, exogenous HMGB1 at optimized concentrations significantly promoted the proliferation, migration, and tube-forming capacity of HUVECs. Consistent with these findings, in vitro aortic ring assays revealed that HMGB1 induced sprouting in rat abdominal aortic tissues, further supporting its pro-angiogenic role. Importantly, administration of 5OMV at varying concentrations after HMGB1 induction effectively inhibited HMGB1-induced HUVEC proliferation and migration. Moreover, 5OMV disrupted both nascent and preformed tubular structures while decelerating the sprouting rate of arterial rings. These collective results conclusively demonstrate 5OMV’s inhibitory effects on HMGB1-mediated angiogenesis.

As the specific receptor for HMGB1, RAGE mediates critical pathological processes. Studies have demonstrated that knockdown of HMGB1 expression in vitro and in vivo blocks the AKT signaling pathway, thereby suppressing gastric cancer cell growth, invasion, and metastasis while inducing apoptosis and cell cycle arrest [25]. Furthermore, HMGB1-RAGE signaling has been implicated in regulating the metastatic invasion of primary liver cancer [26–28]. Notably, Mitola et al. identified HMGB1/RAGE as a potent pro-angiogenic complex in tumor vasculature [29]. Ligand binding to RAGE on endothelial cell surfaces activates the MAPK (MEK/ERK)-NF-κB signaling cascade, triggering inflammatory responses and hepatic injury [30]. Our Western blot (WB) and immunohistochemistry (IHC) analyses revealed that HMGB1 induction upregulated RAGE protein expression in HUVECs, accompanied by increased levels of MEK, p-MEK, ERK, and p-ERK. Pharmacological inhibition of RAGE with FPS-ZM1 [21,22] attenuated HMGB1-induced upregulation of ERK and p-ERK in HUVECs, indicating that HMGB1 activates the downstream MEK/ERK pathway through RAGE to promote angiogenesis. Importantly, 5OMV administration differentially suppressed HMGB1-induced expression of RAGE, MEK, p-MEK, ERK, and p-ERK, with in vivo studies confirming these inhibitory effects. These results collectively demonstrate that 5OMV inhibits HMGB1-driven hepatic angiogenesis through modulation of the RAGE/MEK/ERK signaling pathway.

**Fig 7:**
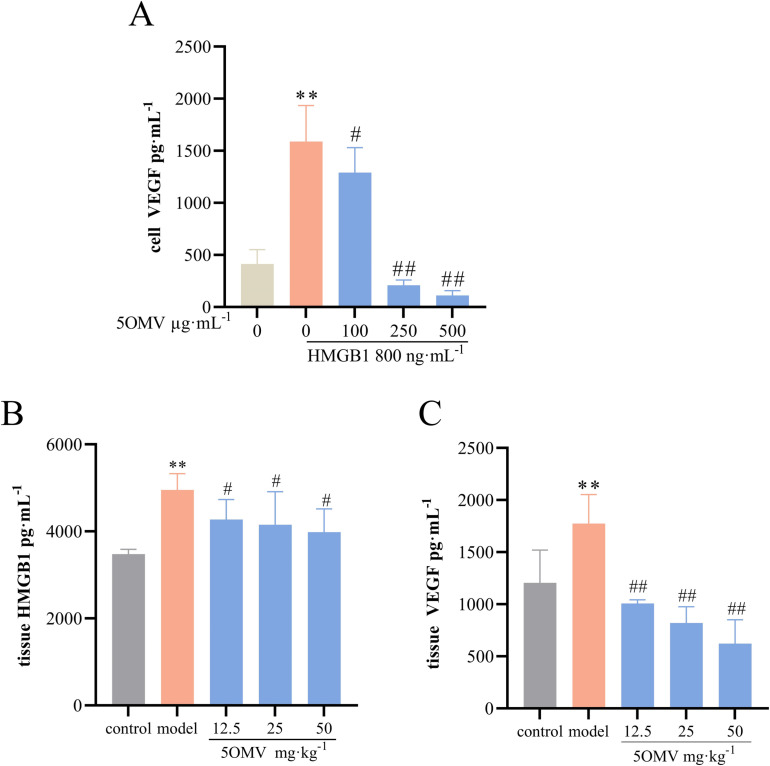
Inhibitory Effects of 5OMV on HMGB1 and VEGF Levels. **(A)** ELISA detection of VEGF levels in HMGB1-induced HUVECs was performed. On day 14 post-administration, liver tumor tissues were excised from hepatocellular carcinoma mice. **(B)** ELISA detection of HMGB1 levels in liver tumor tissues of hepatocellular carcinoma mice was conducted. **(C)** ELISA detection of VEGF levels in liver tumor tissues of hepatocellular carcinoma mice was performed. Data were presented as mean ± SD (n = 3). Error bars represented the standard deviation of three technical replicates. Mean values showing significant differences between the control group and the model group are indicated by *(*P* < 0.05) or ** (*P* < 0.01). Mean values showing significant difference between the model group and the 5OMV-treated group are denoted by ^#^ (*P* < 0.05) or ^##^ (*P* < 0.01). Abbreviation: 5OMV 5-O-Methylvisammioside; HMGB1 High Mobility Group Box 1; HUVECs human umbilical vein endothelial cells; VEGF vascular endothelial growth factor; ELISA enzyme linked immunosorbent assay.

Exogenous HMGB1 has been shown to enhance vascular endothelial growth factor (VEGF) expression in myocardial tissues [31]. In our study, both tumor tissues and HMGB1-stimulated cells exhibited coordinated reductions in VEGF and HMGB1 levels, suggesting a positive correlation between their expression profiles (Fig 7A–C). These findings indicate that 5OMV not only reduces HMGB1 but also attenuates HMGB1-induced VEGF production, potentially exerting broad modulatory effects on the tumor microenvironment. However, the mechanisms underlying 5OMV-mediated HMGB1 suppression, its direct impact on Hepa1–6 cell growth, and subsequent VEGF regulation remain to be fully elucidated. These questions will constitute a key focus of our future investigations to clarify 5OMV’s dual role in microenvironment remodeling and tumor cell proliferation. Interestingly, pharmacological RAGE inhibition using FPS-ZM1 [9] did not diminish 5OMV’s regulatory effects on the RAGE/MEK/ERK pathway but rather demonstrated synergistic activity, though this observation warrants additional validation. While 5OMV demonstrates dual anti-inflammatory and anti-angiogenic activity against hepatocellular carcinoma, the current study is limited by its reliance on a single cell line and animal model. Future work will employ HCC-endothelial cell co-culture systems to better recapitulate the tumor niche, coupled with comprehensive dose-response and chronic toxicity analyses. These approaches will facilitate deeper exploration of 5OMV’s mechanisms in suppressing tumor-associated inflammation and angiogenesis, as well as its translational prospects as a novel therapeutic agent.

Collectively, our findings demonstrate that 5OMV exerts anti-hepatocellular carcinoma effects, potentially through mechanisms involving suppression of HMGB1 levels in hepatic tumor tissues and modulation of the HMGB1/RAGE/MEK/ERK signaling pathway, thereby inhibiting HMGB1-induced angiogenesis. As a natural plant-derived extract, 5OMV exhibits low toxicity coupled with potent anti-angiogenic properties, positioning it as a promising candidate for further development of anti-angiogenic therapeutics in oncology.

## Supporting information

S1 File Sheet 1: Original MTT for Fig 1A, 1C, 1D. Original cell wound closure for Fig 2B. Original cell migration for Fig 2D. Original tube formation numbers for Fig 2F. Original microvessels number for Fig 3B. Original microvascular branches number for Fig 3C. Original tumor weight for Fig 4B. Original tumor inhibition rate for Fig 4C. Original body weight for Fig 4D. Original H-score for Fig 4F. Original Elisa for Fig 7A-7C. Slide 1: Original IHC for Fig 4E. Slide 2: Original IF for Fig 6A. Slide 3: Original IF for Fig 6B. Page1–6: Original western blot for Fig 5B, 5F, 6C.
(ZIP)
