# Supplementary material for: 5-O-Methylvisammioside inhibits HMGB1-induced Angiogenesis of hepatocellular carcinoma through RAGE/MEK/ERK signaling pathway
Source: PLoS One. 2025 May 5;20(5):e0322056. doi: 10.1371/journal.pone.0322056 (PMC12052179; doi:10.1371/journal.pone.0322056)
Supplement: S1 File — Original cell wound closure for Fig 2B. Original cell migration for Fig 2D. Original tube formation numbers for Fig 2F. Original microvessels number for Fig 3B. Original microvascular branches number for Fig 3C. Original tumor weight for Fig 4B. Original tumor inhibition rate for Fig 4C. Original body weight for Fig 4D. Original H-score for Fig 4F. Original Elisa for Fig 7A-7C. Slide 1: Original IHC for Fig 4E. Slide 2: Original IF for Fig 6A. Slide 3: Original IF for Fig 6B. Page1–6: Original western blot for Fig 5B, 5F, 6C. (ZIP) [file pone.0322056.s001.zip › 2-Original IHC-IF.pptx]

## Slide 1
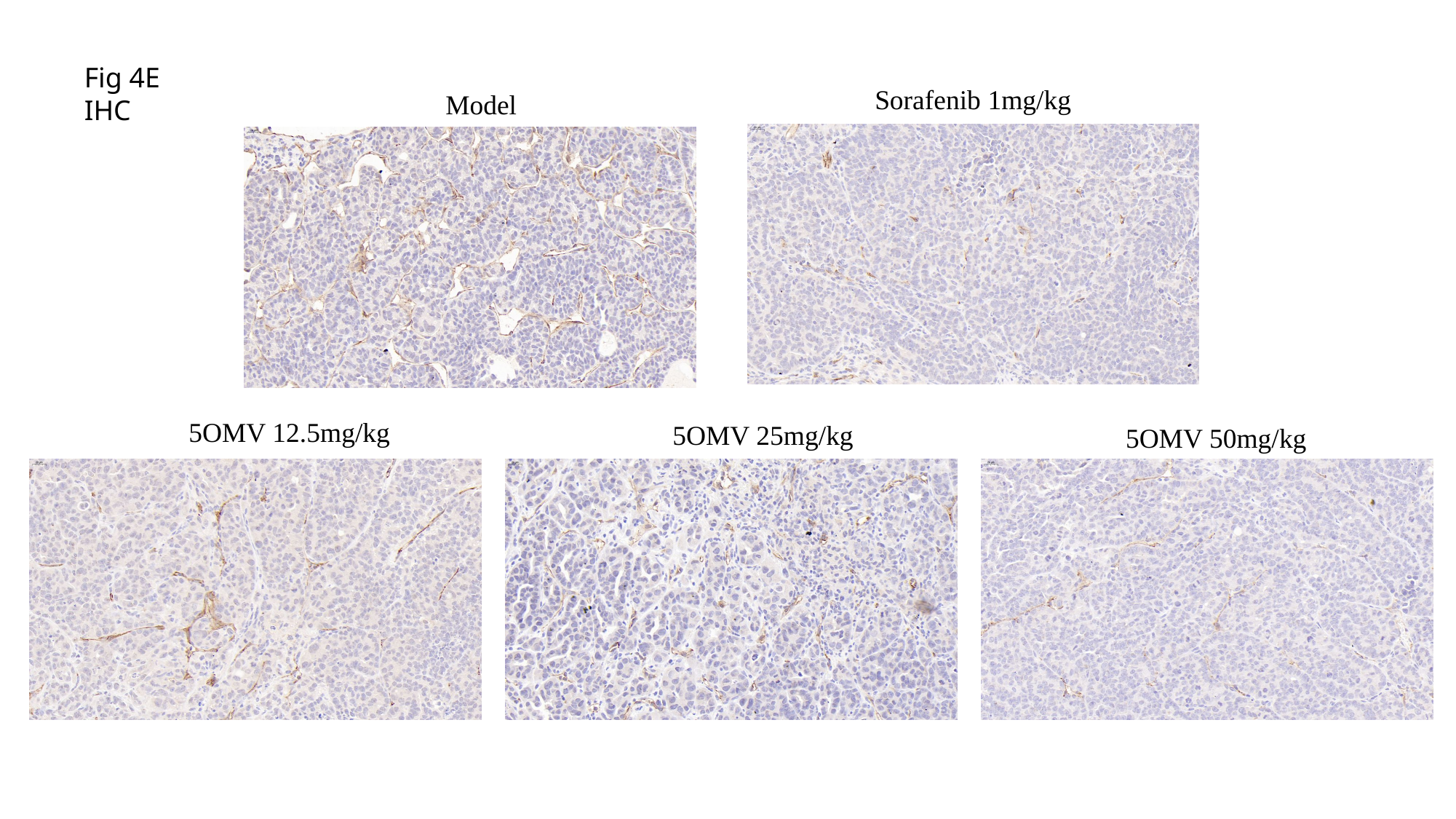

Fig 4E
IHC
Sorafenib 1mg/kg
Model
5OMV 12.5mg/kg
5OMV 25mg/kg
5OMV 50mg/kg

## Slide 2
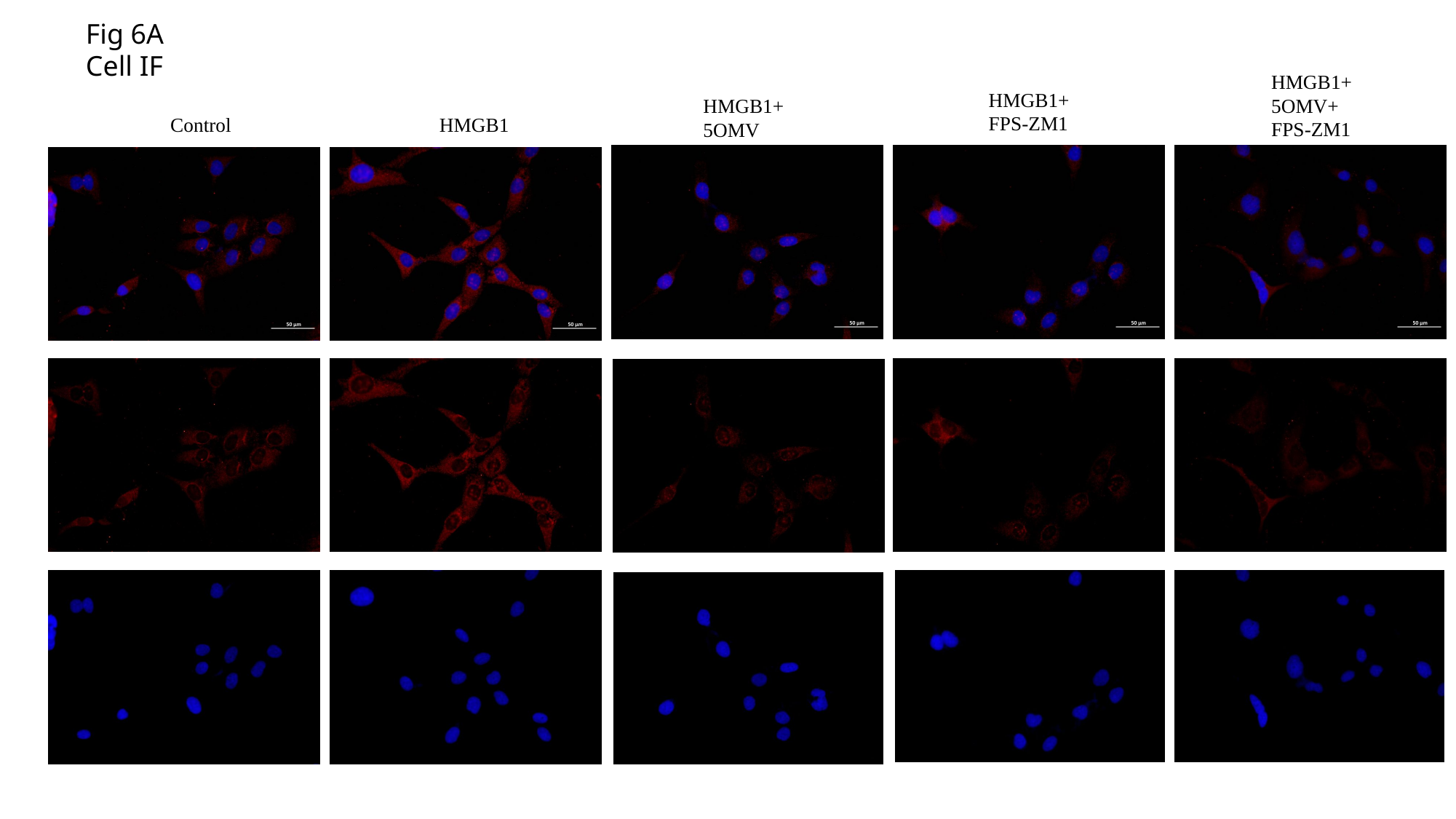

Fig 6A
Cell IF
HMGB1+
5OMV+
FPS-ZM1
HMGB1+
FPS-ZM1
HMGB1+
5OMV
Control
HMGB1

## Slide 3
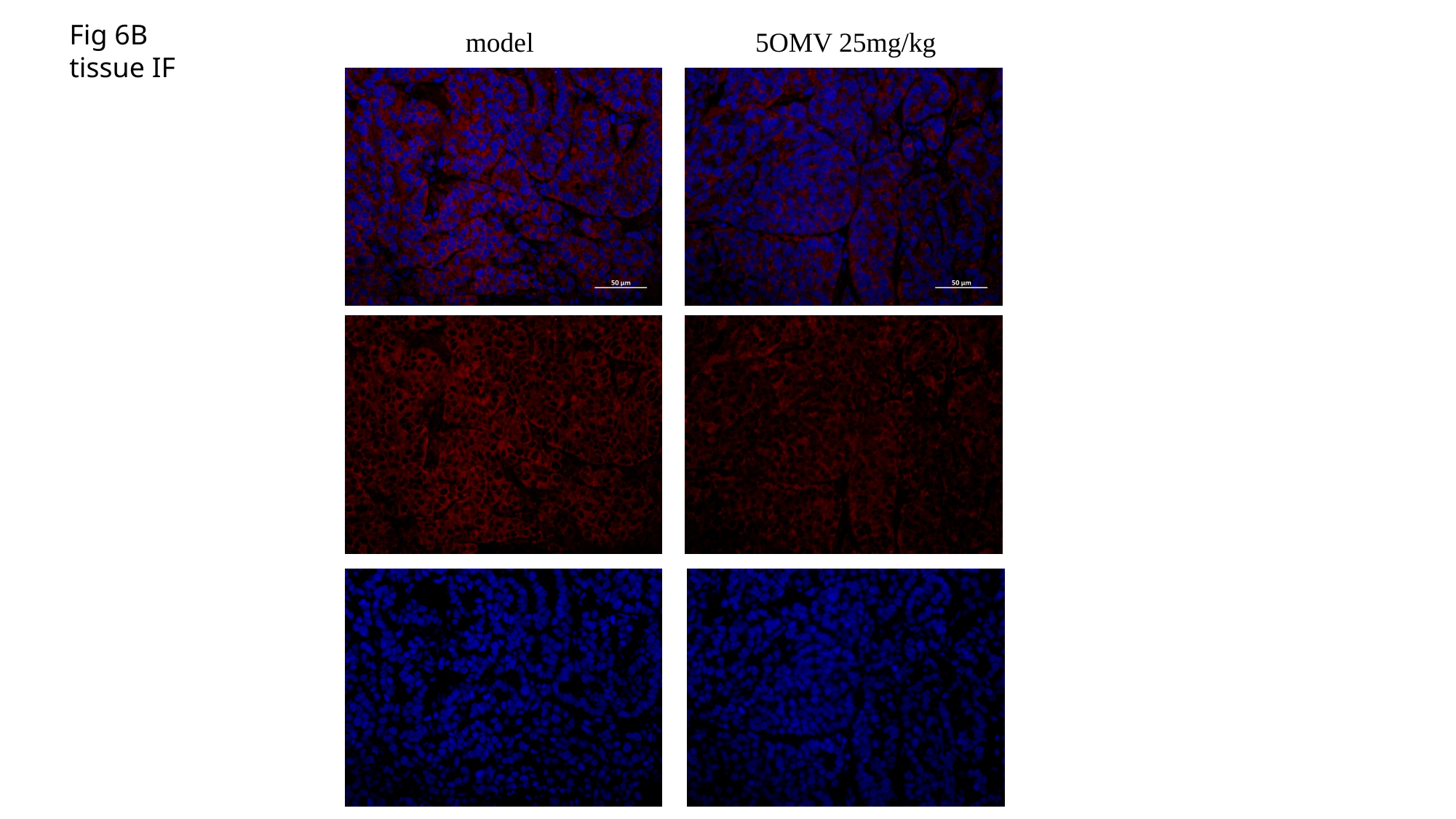

Fig 6B
tissue IF
model
5OMV 25mg/kg
