# Supplementary material for: 5-O-Methylvisammioside inhibits HMGB1-induced Angiogenesis of hepatocellular carcinoma through RAGE/MEK/ERK signaling pathway
Source: PLoS One. 2025 May 5;20(5):e0322056. doi: 10.1371/journal.pone.0322056 (PMC12052179; doi:10.1371/journal.pone.0322056)
Supplement: S1 File — Original cell wound closure for Fig 2B. Original cell migration for Fig 2D. Original tube formation numbers for Fig 2F. Original microvessels number for Fig 3B. Original microvascular branches number for Fig 3C. Original tumor weight for Fig 4B. Original tumor inhibition rate for Fig 4C. Original body weight for Fig 4D. Original H-score for Fig 4F. Original Elisa for Fig 7A-7C. Slide 1: Original IHC for Fig 4E. Slide 2: Original IF for Fig 6A. Slide 3: Original IF for Fig 6B. Page1–6: Original western blot for Fig 5B, 5F, 6C. (ZIP) [file pone.0322056.s001.zip › 3-Original images of Western Blot.pdf]

Figure 5. 5OMV Inhibits RAGE/MEK/ERK Protein Expression in HUVECs

5B Note: The 1, 2, 3, 4, and 5 in the figure represent the control group(Ctrl), model group(Model), low-dose group(L), medium-dose group(M), and high-dose group(H), respectively.

A

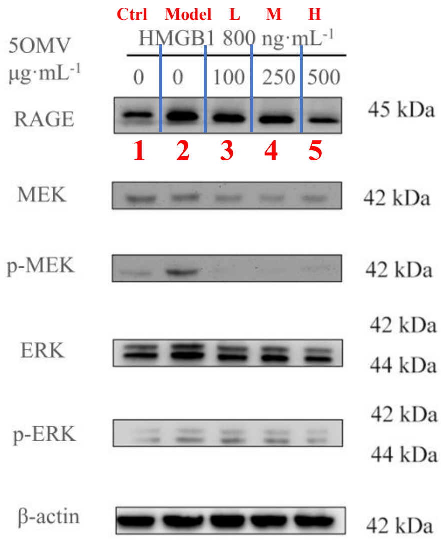

G

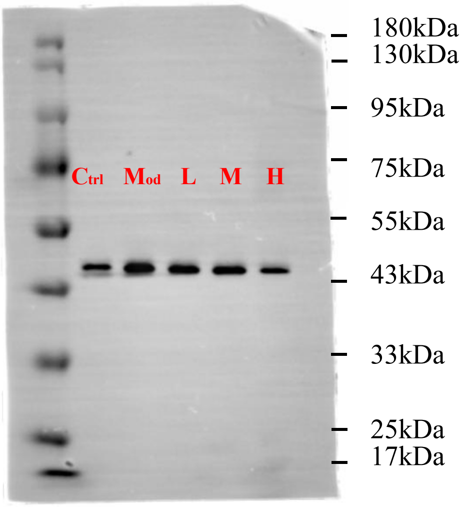

Original Image for Fig5B-RAGE

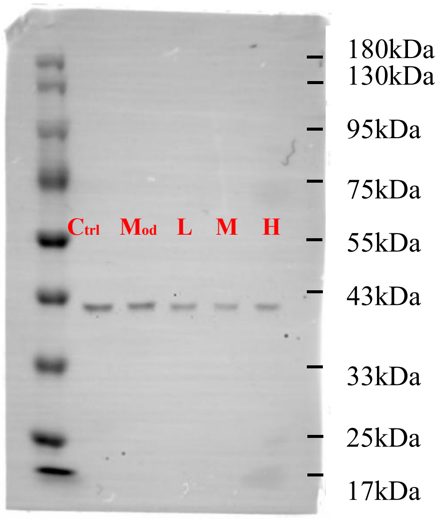

Original Image for Fig5B-MEK

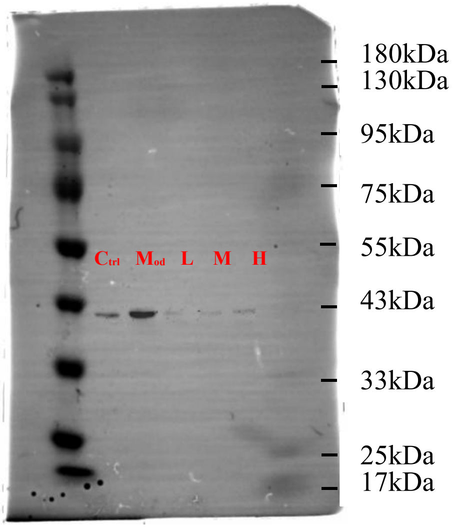

Original Image for Fig5B-p-MEK

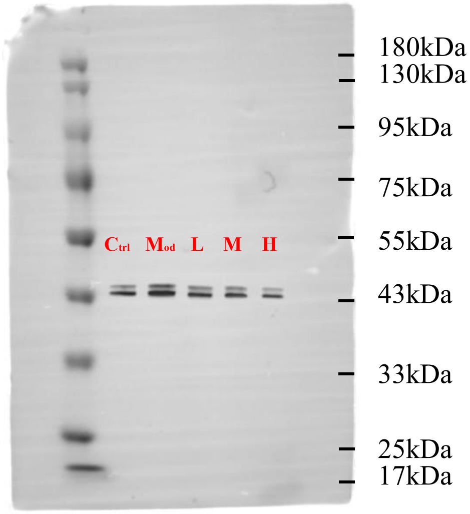

Original Image for Fig5B-ERK

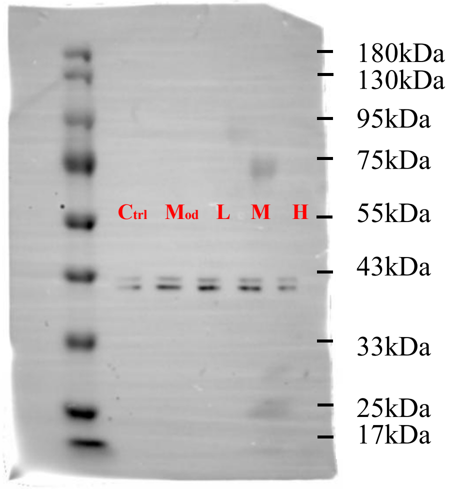

Original Image for Fig5B-p-ERK

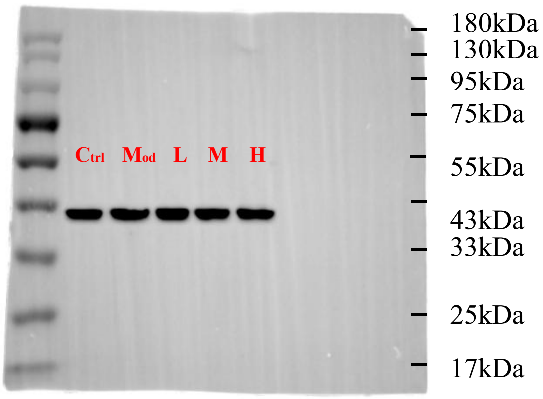

Original Image for Fig5B-β-Actin

5F Note: The 1, 2, 3, 4 in the figure represent the model group(Mod), low-dose group(L), medium-dose group(M), and high-dose group(H), respectively.

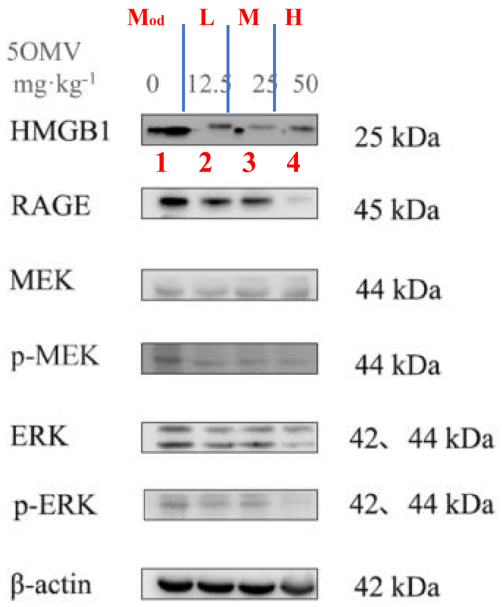

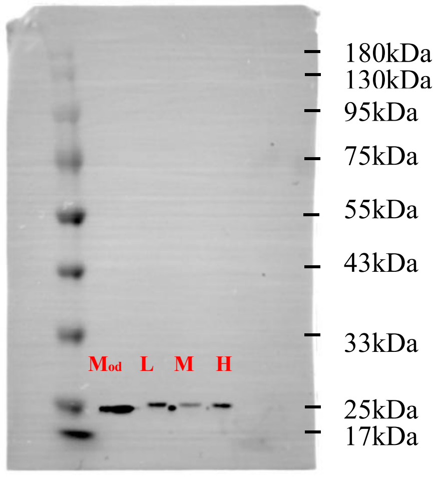

Original Image for Fig5F-HMGB1

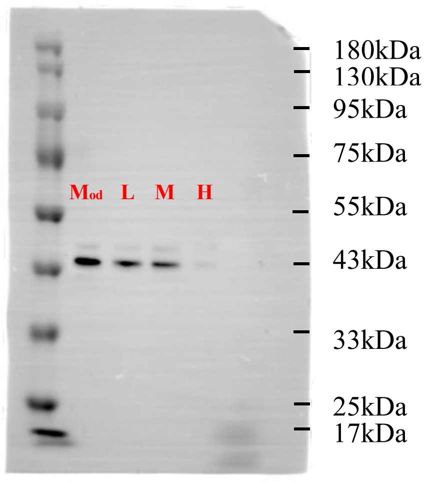

Original Image for Fig5F-RAGE

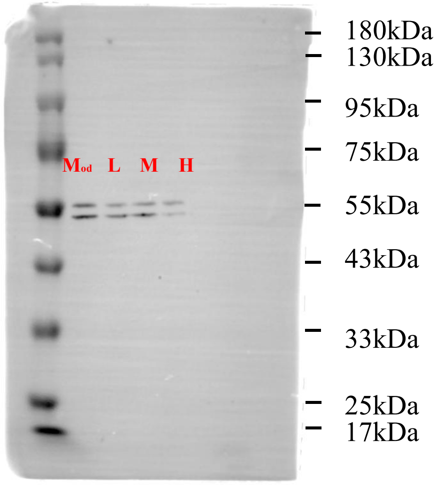

Original Image for Fig5F-ERK

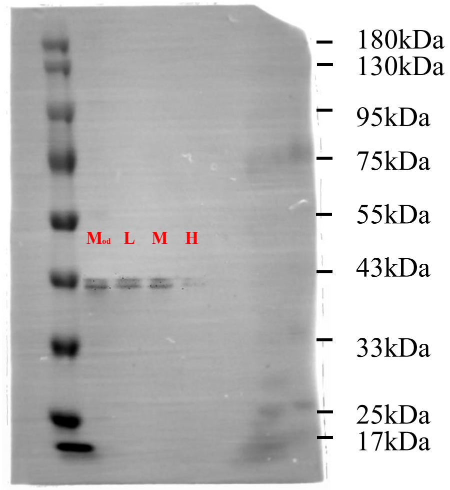

Original Image for Fig5F-p-ERK

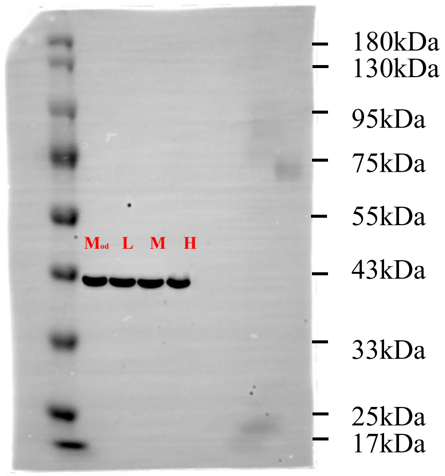

Original Image for Fig5F-β-Actin

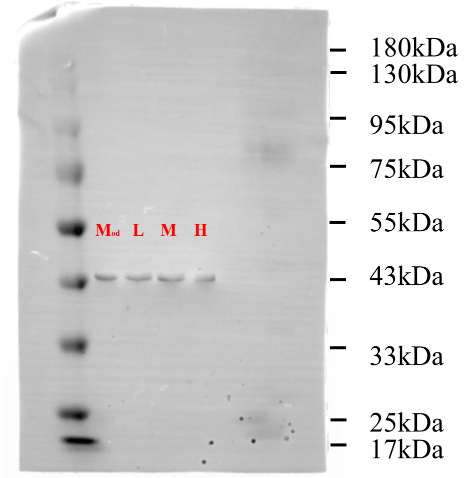

Original Image for Fig5F-MEK

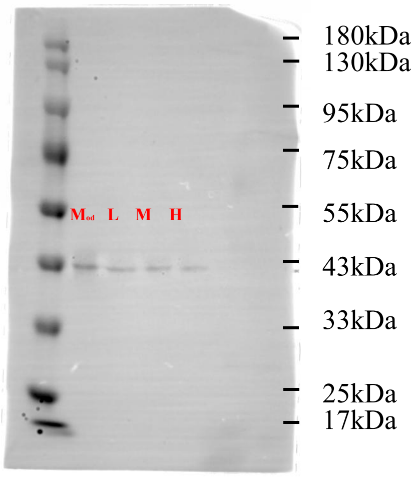

Original Image for Fig5F-p-MEK

Figure 6. RAGE is a Key Target in HMGB1-Induced Tumor Angiogenesis

6C Note: The 1, 2, 3, 4, and 5 in the diagram represent the group order, respectively.

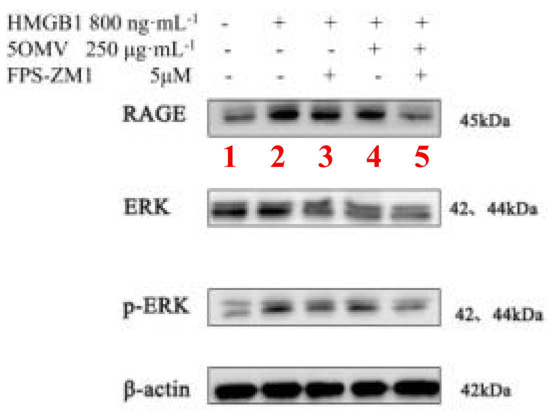

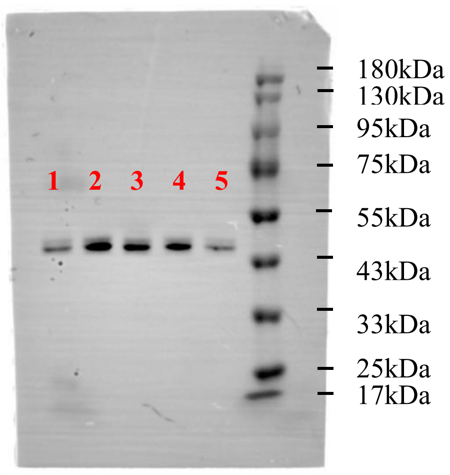

Original Image for Fig6C-RAGE

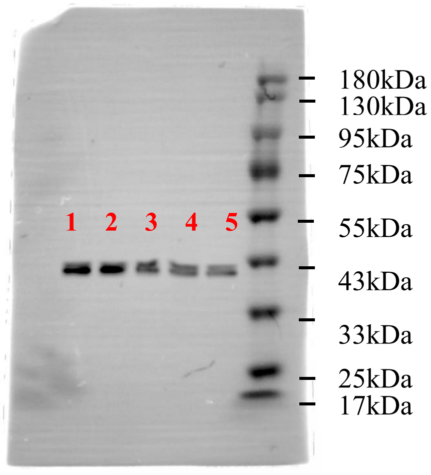

Original Image for Fig6C-ERK

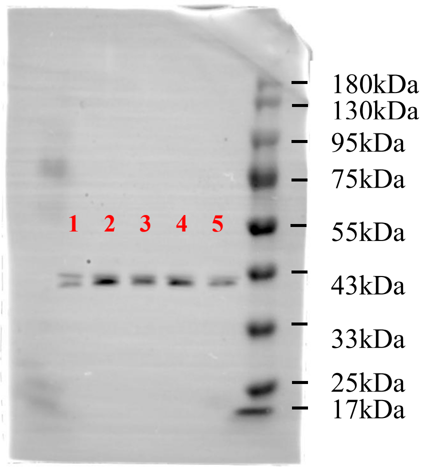

Original Image for Fig6C-p-ERK

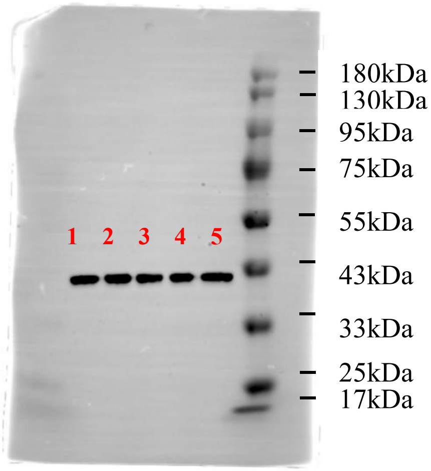

Original Image for Fig6C-β-Actin
